# The Prognostic Value of Gastric Immune Prognostic Index in Gastric Cancer Patients Treated With PD-1/PD-L1 Inhibitors

**DOI:** 10.3389/fphar.2022.833584

**Published:** 2022-06-20

**Authors:** Li Chen, Ruihu Zhao, Hao Sun, Rong Huang, Hongming Pan, Yanjiao Zuo, Lele Zhang, Yingwei Xue, Xingrui Li, Hongjiang Song

**Affiliations:** ^1^ Department of Thyroid and Breast Surgery, Tongji Hospital, Tongji Medical College of Huazhong University of Science and Technology, Wuhan, China; ^2^ Department of Gastrointestinal Surgery, Harbin Medical University Cancer Hospital, Harbin Medical University, Harbin, China

**Keywords:** gastric cancer, gastric immune prognostic index, immune checkpoint inhibitors, derived neutrophil to lymphocyte ratio, lactate dehydrogenase

## Abstract

**Objective:** This study aimed to investigate the prognostic value of the gastric immune prognostic index (GIPI) in gastric cancer patients treated with programmed death 1/programmed death-ligand 1 (PD-1/PD-L1) inhibitors.

**Methods:** This study was conducted to elucidate the role of GIPI using the data from 146 gastric cancer patients treated with PD-1/PD-L1 inhibitors between August 2016 and December 2020 in Harbin Medical University Cancer Hospital. The GIPI calculation was based on dNLR and LDH. Patients were categorized into three groups: 1) GIPI good (LDH ≤250 U/L and dNLR ≤3); 2) GIPI intermediate (LDH >250 U/L and NLR >3); 3) GIPI poor (LDH >250 U/L and dNLR >3). The correlations between GIPI and clinicopathologic characteristics were determined by the Chi-square test or the Fisher’s exact test. The Kaplan–Meier analysis and log-rank test were used to calculate and compare progression-free survival (PFS) and overall survival (OS). The univariate and multivariate Cox proportional hazards regression model was used to detect prognostic and predictive factors of PFS and OS.

**Results:** 146 patients treated with PD-1/PD-L1 inhibitors were included in this study, of which, 72.6% were GIPI good, 23.3% were GIPI intermediate, and 4.1% were GIPI poor. The GIPI was associated with the common blood parameters, including neutrophils and lymphocytes. The multivariate analysis showed that platelet, TNM stage, and treatment were the independent prognostic factors for PFS and OS. Patients with GIPI intermediate/poor were associated with shorter PFS (median: 24.63 vs. 32.50 months; *p* = 0.078) and OS (median: 28.37 months vs. not reached; *p* = 0.033) than those with GIPI good. GIPI intermediate/poor was correlated with shorter PFS and OS than GIPI good, especially in subgroups of patients with ICI treatment and patients with PD-1/PD-L1 positive status.

**Conclusions:** The GIPI correlated with poor outcomes for PD-1/PD-L1 expression status and may be useful for identifying gastric cancer patients who are unlikely to benefit from treatment.

## Introduction

Gastric cancer, the sixth leading cause of cancer-related morbidity and the third leading cause of cancer-related mortality, is one of the commonest gastrointestinal tumors in the world (Sung et al., 2021). Although the incidence rate of gastric cancer has been declining gradually in recent decades, the affected population is always rising worldwide, especially in eastern countries, such as Korea, Japan, Mongolia, and China (Ito et al., 2021). A report indicated that the median survival time of patients with gastric cancer in China during two decades (1980–2000) was 33, 39, and 49 months in 1980, 1990, and 2000s, respectively (Zhang et al., 2011). Despite advances in surgical techniques, chemotherapy, and radiotherapy, the prognosis of gastric cancer has not been significantly improved. Moreover, the death of gastric cancer in China accounts for about 50% of gastric cancer deaths worldwide, with an age-standardized 5-year survival rate of approximately 20% (Zheng et al., 2014; Chen et al., 2016).

Immunotherapy, principally represented by programmed death 1/programmed death ligand 1 (PD-1/PD-L1) inhibitors, has been approved for the treatment of locally advanced, recurrent, metastatic gastric cancer all over the world since September 2017 (Fashoyin-Aje et al., 2019). PD-1 and PD-L1 were momentous immune checkpoint components that essentially regulate the function of tumor-infiltrating lymphocytes and tumor cells. And, PD-1 can negatively regulate the activity of T cells *via* interacting with its ligands PD-L1 expressing on immune cells and tumor cells at some steps of the immune response. The ATTRACTION 02 trial has reported that the median overall survival (OS) was longer in the nivolumab group than in the placebo group. Furthermore, the 12-month OS rate was higher with nivolumab than with placebo in patients with advanced gastric cancer, demonstrating that nivolumab might be a new treatment option for these patients (Kang et al., 2017). The KEYNOTE-061 trial reported that the median OS was 9.1 months with pembrolizumab and 8.3 months with paclitaxel. The trial also reported that the median progression-free survival (PFS) was 1.5 months with pembrolizumab and 4.1 months with paclitaxel, demonstrating that pembrolizumab did not significantly improve OS compared to paclitaxel as second-line therapy for advanced gastric cancer with PD-L1 CPS ≥1 (Shitara et al., 2018). However, even in PD-1/PD-L1 positive populations, the benefits of immunotherapy do not apply to the whole population. This makes the identification of biomarkers in gastric cancer patients likely to respond to immune checkpoint inhibitors (ICIs) therapy—a key step in selecting candidate populations.

The inflammatory process is considered the immune resistance mechanism of cancer patients, promoting cancer growth and metastasis and activating carcinogenic signaling pathways (Gonzalez et al., 2018; McKelvey et al., 2018). In addition, peripheral inflammatory status is related to clinical outcomes in cancer patients. A plethora of routine blood parameters have been studied as potential inflammatory biomarkers in cancer patients, such as neutrophil count, monocyte count, platelet count, lymphocyte count, which are related to the prognosis of several cancer types (Feng et al., 2018; Yakovlev and Klyushin, 2018; Oh et al., 2019; Silvestre-Roig et al., 2019). Novel potential biomarkers, such as neutrophil to lymphocyte ratio, derived neutrophil to lymphocyte ratio [dNLR, absolute neutrophil count/(white blood cell count-absolute neutrophil count)], monocyte to lymphocyte ratio, and platelet to lymphocyte ratio, have been investigated to reflect patients’ immune and inflammatory status in different malignant tumors (Cupp et al., 2020; Gui et al., 2020; Hong et al., 2020; Jakubowska et al., 2020). These ratios, with simple and strong repeatability, are easy to obtain from peripheral blood routine examination.

The prognostic and predictive value of novel inflammatory biomarkers for ICIs is unknown in most tumor types. Recently, Mezquita and colleagues have developed a lung immune prognostic index (LIPI) based on advanced non-small cell lung cancer patients who received ICIs, especially PD-1/PD-L1 inhibitors (Mezquita et al., 2018). The composite index was based on dNLR >3 and LDH > upper limit of normal (ULN) (Mezquita et al., 2018) and characterized into three risk groups: 1) good: dNLR ≤3 and LDH ≤ upper limit of normal (ULN); 2) intermediate: dNLR >3 or LDH > ULN; 3) poor: dNLR >3 and LDH > ULN. The authors also observed that the LIPI was related to the clinical outcome with ICI-treated immunotherapy but not cytotoxic chemotherapy (CCT). This might help doctors determine which patients can benefit from treatment. However, the correlation of gastric immune prognostic index (GIPI) with PD-1/PD-L1 inhibitors outcomes has not been studied in gastric cancer patients. Therefore, we performed an exploratory retrospective analysis to investigate the prognostic value of GIPI in gastric cancer patients treated with PD-1/PD-L1 inhibitors.

## Materials and Methods

### Patients’ Selection

Institutional review board approval was acquired to review medical records at Harbin Medical University Cancer Hospital. All patient data accessed complied with relevant data protection and privacy regulations. All processes performed in the study were conducted in accordance with the standards of the institutional research committee and with the declaration of Goodyear et al. (2007) as well as its later amendments or comparable ethical standards. Informed consent was waived by the Ethics Committee of Harbin Medical University Cancer Hospital due to the retrospective nature of this study. Between August 2016 and December 2020, 146 patients with gastric cancer treated with PD-1/PD-L1 inhibitors at Harbin Medical University Cancer Hospital were included. We collected and searched the clinical data by electronic medical records. The inclusion criteria were as follows: 1) patients who were diagnosed with gastric cancer; 2) patients receiving PD-1/PD-L1 inhibitors or chemotherapy; and 3) Eastern Cooperative Oncology Group performance status: 0–2. The exclusion criteria were as follows: 1) absence of pretreatment blood test results; 2) autoimmune disease or systemic immunosuppression; and 3) absence of efficacy assessment.

### Calculation of Gastric Immune Prognostic Index

The GIPI, which comprises two factors, was based on dNLR and serum LDH levels. Information on complete blood cell counts with differential counts and LDH levels within 7 days before treatment was extracted. The cutoff value of LDH was determined based on ULN (250 IU/L). The cutoff value of dNLR was set at >3, as reported by Mezquita et al. (2018). Patients were categorized into three groups: 1) GIPI good (LDH ≤250 U/L and dNLR ≤3); 2) GIPI intermediate (LDH >250 U/L and NLR >3); and 3) GIPI poor (LDH >250 U/L and dNLR >3).

### Immunohistochemistry for Programmed Death 1/Programmed Death-Ligand 1

The gastric cancer tissues were fixed with methanol, embedded in paraffin, sectioned, and performed immunohistochemical analyses. PD-1/PD-L1 expression was analyzed on tumor cells using immunohistochemistry, according to the instructions of the manufacturer. The expression of at least 1% was considered positive (Zayac and Almhanna, 2020).

### Follow-Up

All enrolled patients were routinely followed-up by telephone, inpatient, and outpatient. Follow-up assessments included laboratory tests, physical examination, multi-slice CT, gastroscopy, and some other examinations as it fits. PFS was calculated from the date of the first immunotherapy administration to the date of disease progression or death due to any cause. OS was calculated from the date of the first immunotherapy administration to the date of death from any cause. The date of the last follow-up in this study was November 2021.

### Statistical Analysis

The clinical characteristics of the patients were presented as absolute values and percentages (%). Discrete variables were compared using the Chi-square test or Fishers exact test, and the Student’s t-test was used for continuous variables. The Kaplan-Meier curves were used to evaluate OS and PFS, and the differences were evaluated by a log-rank test. The univariate and multivariate Cox proportional hazards regression model was used to evaluate the independent prognostic factors. The hazard ratio with its 95% confidence interval was estimated using the univariate and multivariate Cox proportional hazards regression model. All *p* values were from two-sided tests and were considered statistically significant at two-tailed *p* < 0.05. All statistical analyses were performed using the R (version 3.6.0; Vienna, Austria. URL: http://www.R-project.org/), SPSS software (version 17.0; SPSS Inc., Chicago, IL, United States), and GraphPad Prism software (version 8.0; GraphPad Inc., La Jolla, CA, United States).

## Results

### Patient Characteristics

According to the GIPI, 106 (72.6%), 34 (23.3%), and 6 (4.1%) patients were allocated to the GIPI good, GIPI intermediate, and GIPI poor groups, respectively. Due to the small number of patients in the poor GIPI group, all patients were divided into two groups: GIPI good group with 106 (72.6%) patients and GIPI intermediate/poor group with 40 (37.4%) patients. The clinical characteristics of the enrolled patients are summarized in Table 1. There were 102 males and 44 females diagnosed with gastric cancer in the study population. The median age was 59 years (range: 34–82 years). The median BMI was 21.55 (range: 15.15–34.21). In the light of ABO blood type, A type was 48 cases (32.9%), B type was 38 cases (26.0%), O type was 49 cases (33.6%), and AB type was 11 cases (7.5%). Based on the 8th edition of the TNM classification, 10 (6.8%), 14 (9.6%), 39 (26.7%), and 83 (56.8%) gastric cancer patients were classified as stage I, II, III, and IV, respectively. GIPI was associated with surgery (*p* = 0.022). The detailed information is shown in Table 1.

**TABLE 1:** The clinical characteristics of all enrolled patients.

n	Level	GIPI good 106	GIPI intermediate/poor 40	*p*
Sex (%)	Male	74 (69.8)	28 (70.0)	1.000
	Female	32 (30.2)	12 (30.0)	
Age [median (IQR)]		61.0 (53.3, 66.0)	57.5 (50.8–63.3)	0.200
Age (%)	<59	46 (43.4)	22 (55.0)	0.286
	≥59	60 (56.6)	18 (45.0)	
Profession (%)	Mental worker	19 (17.9)	6 (15.0)	0.863
	Manual worker	87 (82.1)	34 (85.0)	
BMI (%)	<21.55	54 (50.9)	19 (47.5)	0.853
	≥21.55	52 (49.1)	21 (52.5)	
Drinking water (%)	Deep well water	72 (67.9)	27 (67.5)	1.000
	Surface water	34 (32.1)	13 (32.5)	
Stomach ache (%)	No	84 (79.2)	36 (90.0)	0.203
	Yes	22 (20.8)	4 (10.0)	
Abdominal distention and pain (%)	No	96 (90.6)	40 (100.0)	0.100
	Yes	10 (9.4)	0 (0.0)	
Black stool (%)	No	101 (95.3)	39 (97.5)	0.893
	Yes	5 (4.7)	1 (2.5)	
Weight loss (%)	No	95 (89.6)	37 (92.5)	0.832
	Yes	11 (10.4)	3 (7.5)	
Fatigue (%)	No	99 (93.4)	40 (100.0)	0.218
	Yes	7 (6.6)	0 (0.0)	
Sour regurgitation (%)	No	99 (93.4)	39 (97.5)	0.573
	Yes	7 (6.6)	1 (2.5)	
ABO blood type (%)	A	34 (32.1)	14 (35.0)	0.565
	B	25 (23.6)	13 (32.5)	
	O	38 (35.8)	11 (27.5)	
	AB	9 (8.5)	2 (5.0)	
Surgery (%)	No	37 (34.9)	23 (57.5)	0.022
	Yes	69 (65.1)	17 (42.5)	
Primary tumor site (%)	Upper 1/3	16 (15.1)	5 (12.5)	0.501
	Middle 1/3	32 (30.2)	13 (32.5)	
	Low 1/3	48 (45.3)	21 (52.5)	
	Whole	10 (9.4)	1 (2.5)	
Borrmann type (%)	Borrmann I	2 (1.9)	1 (2.5)	0.132
	Borrmann II	5 (4.7)	1 (2.5)	
	Borrmann III	55 (51.9)	12 (30.0)	
	Borrmann IV	7 (6.6)	3 (7.5)	
	Unknown	37 (34.9)	23 (57.5)	
Tumor size (%)	<50 mm	33 (31.1)	8 (20.0)	0.174
	≥50 mm	25 (23.6)	7 (17.5)	
	Unknown	48 (45.3)	25 (62.5)	
Differentiation (%)	Poorly differentiated	64 (60.4)	31 (77.5)	0.165
	Moderately differentiated	32 (30.2)	7 (17.5)	
	Well differentiated	1 (0.9)	1 (2.5)	
	Unknown	9 (8.5)	1 (2.5)	
Pathology (%)	Adenocarcinoma	65 (61.3)	32 (80.0)	0.185
	Mucinous carcinoma	5 (4.7)	0 (0.0)	
	Signet ring cell carcinoma	9 (8.5)	3 (7.5)	
	Mixed carcinoma	16 (15.1)	4 (10.0)	
	Others	11 (10.4)	1 (2.5)	
TNM stage (%)	I	7 (6.6)	3 (7.5)	0.223
	II	13 (12.3)	1 (2.5)	
	III	30 (28.3)	9 (22.5)	
	IV	56 (52.8)	27 (67.5)	
Lauren type (%)	Intestinal	27 (25.5)	6 (15.0)	0.426
	Diffuse	16 (15.1)	5 (12.5)	
	Mixed	17 (16.0)	6 (15.0)	
	Unknown	46 (43.4)	23 (57.5)	
PD-1 (%)	Negative	49 (46.2)	16 (40.0)	0.715
	Positive	12 (11.3)	4 (10.0)	
	Unknown	45 (42.5)	20 (50.0)	
PD-L1 (%)	Negative	33 (31.1)	9 (22.5)	0.566
	Positive	28 (26.4)	11 (27.5)	
	Unknown	45 (42.5)	20 (50.0)	
Treatment (%)	ICIs	59 (55.7)	30 (75.0)	0.052
	Chemotherapy	47 (44.3)	10 (25.0)	

BMI, body mass index; PD-1, programmed death 1; PD-L1, programmed death-ligand 1; ICIs, PD-1/PD-L1, inhibitors.

### Blood Parameters

The median of the white blood cell (WBC), neutrophil (NEU), lymphocyte (LYM), monocyte (MONO), eosinophils (EOS), basophil (BASO), red blood cell (RBC), and platelet (PLT) counts were 6.44 × 10^9^/L, 3.82 × 10^9^/L, 1.70 × 10^9^/L, 0.48 × 10^9^/L, 0.09 × 10^9^/L, 0.02 × 10^9^/L, 4.34 × 10^12^/L, and 232 × 10^9^/L, respectively. GIPI was associated with LDH (*p* < 0.001), dNLR (*p* < 0.001), WBC (*p* = 0.016), NEU (*p* = 0.002), LYM (*p* < 0.001), MONO (*p* = 0.021), BASO (*p* = 0.023), and RBC (*p* = 0.032), respectively. The detailed information is shown in Table 2.

**TABLE 2:** The blood parameters of all enrolled patients.

n	Level	GIPI good 106	GIPI intermediate/poor 40	*p*
LDH (%)	<250	106 (100.0)	18 (45.0)	<0.001
	≥250	0 (0.0)	22 (55.0)	
dNLR (%)	<3.0	106 (100.0)	16 (40.0)	<0.001
	≥3.0	0 (0.0)	24 (60.0)	
WBC (%)	<6.44	60 (56.6)	13 (32.5)	0.016
	≥6.44	46 (43.4)	27 (67.5)	
NEU (%)	<3.82	62 (58.5)	11 (27.5)	0.002
	≥3.82	44 (41.5)	29 (72.5)	
LYM (%)	<1.70	42 (39.6)	31 (77.5)	<0.001
	≥1.70	64 (60.4)	9 (22.5)	
MONO (%)	<0.48	59 (55.7)	13 (32.5)	0.021
	≥0.48	47 (44.3)	27 (67.5)	
EOS (%)	<0.09	43 (40.6)	24 (60.0)	0.055
	≥0.09	63 (59.4)	16 (40.0)	
BASO (%)	<0.02	19 (17.9)	15 (37.5)	0.023
	≥0.02	87 (82.1)	25 (62.5)	
RBC (%)	<4.34	46 (43.4)	26 (65.0)	0.032
	≥4.34	60 (56.6)	14 (35.0)	
PLT (%)	<232.0	48 (45.3)	24 (60.0)	0.161
	≥232.0	58 (54.7)	16 (40.0)	

LDH, lactate dehydrogenase; dNLR, derived neutrophil to lymphocyte ratio; WBC, white blood cell; NEU, neutrophils; LYM, lymphocyte; MONO, monocyte; EOS, eosinophils; BASO, basophil; RBC, red blood cell; PLT, platelet.

### Univariate and Multivariate Analysis for Progression-Free Survival and Overall Survival

The univariate analysis showed that PLT, GIPI, LDH, radical resection, surgery, TNM stage, Lauren type, treatment, PD-1, and PD-L1 were associated with the prognosis of patients with gastric cancer for PFS. However, the multivariate analysis indicated that PLT, TNM stage, and treatment were the independent prognostic factors for PFS (Table 3). Furthermore, the univariate analysis indicated that PLT, GIPI, LDH, radical resection, surgery, Borrmann type, Lauren type, treatment, PD-1, and PD-L1 were associated with the prognosis of patients with gastric cancer for OS. Nevertheless, the multivariate analysis showed that PLT, TNM stage, and treatment were the independent prognostic factors for OS (Table 3).

**TABLE 3:** Univariate and multivariate Cox hazard analysis of biomarkers for progression-free survival (PFS) and overall survival (OS).

Parameters	Level	PFS			*p*-value	OS			*p*-value
		Univariate analysis	*p*-value	Multivariate analysis		Univariate analysis	*p*-value	Multivariate analysis	
		Hazard ratio (95%CI)		Hazard ratio (95%CI)		Hazard ratio (95%CI)		Hazard ratio (95%CI)	
Sex	Male		0.843				0.768		
	Female	1.055 (0.620–1.798)				1.084 (0.636–1.846)			
Age (year)	<59		0.617				0.603		
	≥59	0.881 (0.535–1.45)				0.876 (0.532–1.442)			
BMI	<21.55		0.690				0.588		
	≥21.55	0.903 (0.549–1.487)				0.871 (0.529–1.434)			
ABO blood type	A + B		0.135				0.182		
	O + AB	1.463 (0.889–2.409)				1.404 (0.853–2.311)			
WBC (10^9^/L)	<6.44		0.644				0.664		
	≥6.44	0.889 (0.539–1.467)				0.895 (0.543–1.477)			
NEU (10^9^/L)	<3.82		0.663				0.725		
	≥3.82	1.118 (0.678–1.841)				1.094 (0.664–1.803)			
LYM (10^9^/L)	<1.70		0.073				0.094		
	≥1.70	0.630 (0.380–1.045)				0.649 (0.392–1.076)			
MONO (10^9^/L)	<0.48		0.661				0.691		
	≥0.48	0.894 (0.541–1.476)				0.903 (0.547–1.492)			
EOS (10^9^/L)	<0.09		0.876				0.780		
	≥0.09	0.961 (0.584–1.583)				0.931 (0.566–1.534)			
BASO (10^9^/L)	<0.02		0.444				0.324		
	≥0.02	1.279 (0.681–2.403)				1.373 (0.731–2.580)			
RBC (10^12^/L)	<4.34		0.676				0.783		
	≥4.34	1.112 (0.675–1.833)				1.073 (0.651–1.767)			
PLT (10^9^/L)	<232.0		0.037		0.004		0.036		0.005
	≥232.0	0.581 (0.348–0.968)		0.425 (0.237–0.761)		0.579 (0.347–0.966)		0.430 (0.237–0.780)	
GIPI	Good		0.036		0.555		0.035		0.335
	Intermediate/poor	1.747 (1.036–2.945)		0.7627 (0.31–1.876)		1.751 (1.039–2.951)		0.6348 (0.252–1.599)	
LDH (U/L)	<250		0.034		0.335		0.027		0.147
	≥250	1.942 (1.052–3.586)		1.657 (0.593–4.626)		1.997 (1.081–3.689)		2.214 (0.757–6.481)	
dNLR	<3.0		0.300				0.317		
	≥3.0	1.398 (0.743–2.630)				1.38 (0.734–2.596)			
Radical resection	R0		0.001		0.062		0.000		0.056
	R1+R2+unknown	2.568 (1.499–4.401)		2.509 (0.954–6.598)		2.800 (1.631–4.807)		2.614 (0.978–6.989)	
Surgery	Yes		0.012		0.425		0.006		0.536
	No	1.942 (1.155–3.263)		0.701 (0.292–1.680)		2.093 (1.242–3.526)		1.608 (0.358–7.232)	
Primary tumor site	Low 1/3		0.850				0.952		
	Upper 1/3 + middle 1/3 + whole	0.953 (0.579–1.569)				0.985 (0.598–1.622)			
Borrmann type	Borrmann I + II		0.065				0.040		0.145
	Borrmann III + IV + unknown	1.545 (0.973–2.455)				1.636 (1.023–2.616)		0.431 (0.139–1.337)	
Tumor size	<50 mm		0.440				0.377		
	≥50 mm + unknown	1.121 (0.839–1.497)				1.140 (0.853–1.523)			
Differentiation	Poorly		0.592				0.488		
	Moderately + well + unknown	0.889 (0.577–1.368)				0.857 (0.553–1.327)			
Pathology	Adenocarcinoma		0.106				0.094		
	Others^a^	1.363 (0.936–1.983)				1.380 (0.947–2.011)			
TNM stage	I + II		0.002		0.009		0.001		0.005
	III + IV	6.530 (2.037–20.940)		6.432 (1.600–25.790)		6.605 (2.060–21.180)		7.711 (1.850–32.100)	
Lauren type	Intestinal		0.005		0.899		0.002		0.921
	Diffuse + mixed + unknown	1.390 (1.105–1.747)		0.976 (0.671–1.420)		1.426 (1.133–1.796)		0.981 (0.665–1.446)	
Treatment	ICIs		0.014		0.007		0.010		0.004
	Chemotherapy	4.354 (1.354–14.000)		9.761 (1.840–51.730)		4.661 (1.448–15.000)		12.520 (2.270–69.040)	
PD-1	Negative + unknown		0.000		0.972		0.000		0.900
	Positive	1.677 (1.260–2.232)		1.017 (0.408–2.532)		1.694 (1.272–2.256)		0.944 (0.386–2.311)	
PD-L1	Negative + unknown		0.001		0.816		0.000		0.538
	Positive	1.808 (1.286–2.541)		1.138 (0.383–3.379)		1.840 (1.311–2.582)		1.402 (0.479–4.109)	

a Others: mucinous carcinoma, signet ring cell carcinoma, mixed carcinoma, unknown.

BMI, body mass index; PD-1, Programmed death 1; PD-L1, Programmed death-ligand 1; ICIs, PD-1/PD-L1, inhibitors; LDH, lactate dehydrogenase; GIPI, gastric immune prognostic index; dNLR, derived neutrophil to lymphocyte ratio; WBC, white blood cell; NEU, neutrophils; LYM, lymphocyte; MONO, monocyte; EOS, eosinophils; BASO, basophil; RBC, red blood cell; PLT, platelet.

### Survival Outcomes With Derived Neutrophil to Lymphocyte Ratio, Lactate Dehydrogenase, and Gastric Immune Prognostic Index

**FIGURE 1:**
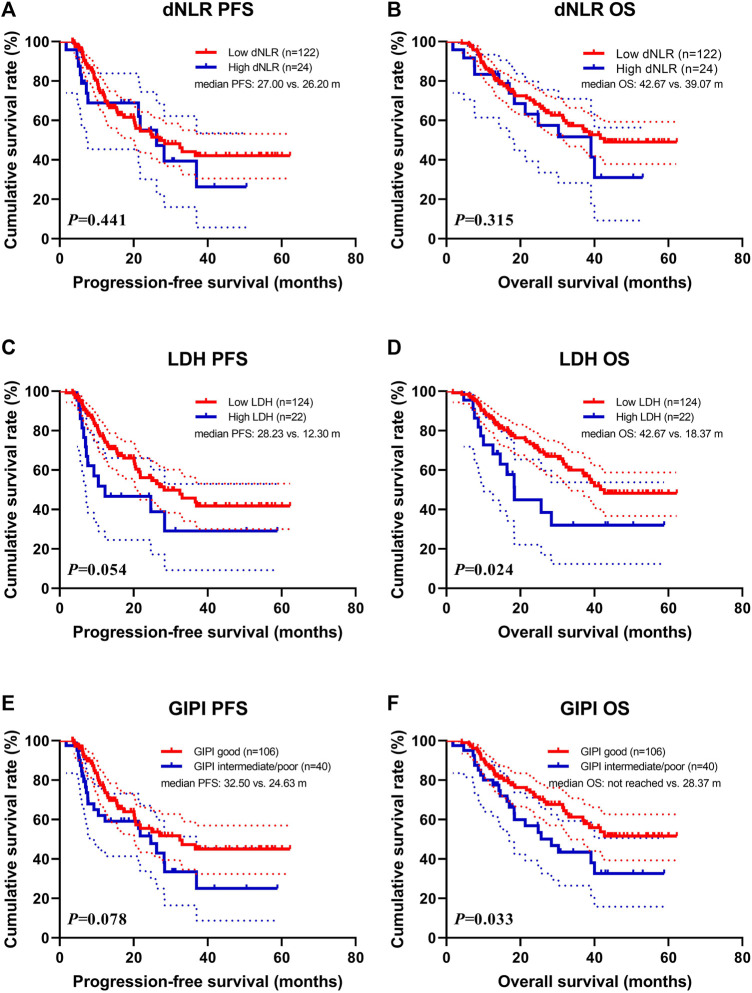
Survival according to dNLR, LDH, and GIPI groups for **(A)** progression-free survival (PFS) by dNLR; **(B)** overall survival (OS) by dNLR; **(C)** PFS by LDH; **(D)** OS by LDH; **(E)** PFS by GIPI; **(F)** OS by GIPI.

Patients with high dNLR were associated with shorter PFS (median: 26.20 vs. 27.00 months; *p* = 0.441) and OS (median: 39.07 vs. 42.67 months; *p* = 0.315) than those with high dNLR (Figures 1A,B). Patients with high LDH were associated with shorter PFS (median: 12.30 vs. 28.23 months; *p* = 0.054) and OS (median: 18.37 vs. 42.67 months; *p* = 0.024) than those with low LDH (Figures 1C,D). Patients with GIPI intermediate/poor were associated with shorter PFS (median: 24.63 vs. 32.50 months; *p* = 0.078) and OS (median: 28.37 months vs. not reached; *p* = 0.033) than those with GIPI good (Figures 1E,F).

### Treatment (Immune Checkpoint Inhibitors and Chemotherapy)

In this study, 89 patients received PD-1/PD-L1 inhibitors treatment (named ICIs group), and 57 patients received chemotherapy (including targeted therapy) treatment (named chemotherapy group). Baseline demographics and disease characteristics are shown in Table 4. Between the two groups, statistically significant differences were found in surgery (*p* < 0.001), Borrmann type (*p* < 0.001), tumor size (*p* = 0.046), differentiation (*p* = 0.001), TNM stage (*p* < 0.001), Lauren type (*p* < 0.001), PD-1 (*p* < 0.001), and PD-L1 (*p* < 0.001). Among the blood parameters, statistically significant differences were found in dNLR (*p* = 0.026), WBC (*p* = 0.042), NEU (*p* = 0.018), and MONO (*p* = 0.012) (Table 5).

**TABLE 4:** The clinical characteristics for treatment (ICIs and chemotherapy).

n	Level	ICIs 89	Chemotherapy 57	*p*
Sex (%)	Male	65 (73.0)	37 (64.9)	0.391
	Female	24 (27.0)	20 (35.1)	
Age [median (IQR)]		59.0 (53.0–66.0)	60.0 (49.0–66.0)	0.727
Age (%)	<59	42 (47.2)	26 (45.6)	0.987
	≥59	47 (52.8)	31 (54.4)	
Profession (%)	Mental worker	17 (19.1)	8 (14.0)	0.570
	Manual worker	72 (80.9)	49 (86.0)	
BMI (%)	<21.55	40 (44.9)	33 (57.9)	0.175
	≥21.55	49 (55.1)	24 (42.1)	
Drinking water (%)	Deep well water	63 (70.8)	36 (63.2)	0.435
	Surface water	26 (29.2)	21 (36.8)	
Stomachache (%)	No	70 (78.7)	50 (87.7)	0.240
	Yes	19 (21.3)	7 (12.3)	
Abdominal distention and pain (%)	No	83 (93.3)	53 (93.0)	1.000
	Yes	6 (6.7)	4 (7.0)	
Black stool (%)	No	84 (94.4)	56 (98.2)	0.472
	Yes	5 (5.6)	1 (1.8)	
Weight loss (%)	No	78 (87.6)	54 (94.7)	0.257
	Yes	11 (12.4)	3 (5.3)	
Fatigue (%)	No	83 (93.3)	56 (98.2)	0.328
	Yes	6 (6.7)	1 (1.8)	
Sour regurgitation (%)	No	83 (93.3)	55 (96.5)	0.642
	Yes	6 (6.7)	2 (3.5)	
ABO blood type (%)	A	33 (37.1)	15 (26.3)	0.205
	B	25 (28.1)	13 (22.8)	
	O	24 (27.0)	25 (43.9)	
	AB	7 (7.9)	4 (7.0)	
Surgery (%)	Yes	38 (42.7)	48 (84.2)	<0.001
	No	51 (57.3)	9 (15.8)	
Primary tumor site (%)	Upper 1/3	14 (15.7)	7 (12.3)	0.902
	Middle 1/3	28 (31.5)	17 (29.8)	
	Low 1/3	41 (46.1)	28 (49.1)	
	Whole	6 (6.7)	5 (8.8)	
Borrmann type (%)	Borrmann I	2 (2.2)	1 (1.8)	<0.001
	Borrmann II	4 (4.5)	2 (3.5)	
	Borrmann III	23 (25.8)	44 (77.2)	
	Borrmann IV	9 (10.1)	1 (1.8)	
	Unknown	51 (57.3)	9 (15.8)	
Tumor size (%)	<50 mm	19 (21.3)	22 (38.6)	0.046
	≥50 mm	19 (21.3)	13 (22.8)	
	Unknown	51 (57.3)	22 (38.6)	
Differentiation (%)	Poorly differentiated	63 (70.8)	32 (56.1)	0.001
	Moderately differentiated	16 (18.0)	23 (40.4)	
	Well differentiated	0 (0.0)	2 (3.5)	
	Unknown	10 (11.2)	0 (0.0)	
Pathology (%)	Adenocarcinoma	60 (67.4)	37 (64.9)	0.357
	Mucinous carcinoma	2 (2.2)	3 (5.3)	
	Signet ring cell carcinoma	6 (6.7)	6 (10.5)	
	Mixed carcinoma	11 (12.4)	9 (15.8)	
	Others	10 (11.2)	2 (3.5)	
TNM stage (%)	I	3 (3.4)	7 (12.3)	<0.001
	II	5 (5.6)	9 (15.8)	
	III	15 (16.9)	24 (42.1)	
	IV	66 (74.2)	17 (29.8)	
Lauren type (%)	Intestinal	14 (15.7)	19 (33.3)	<0.001
	Diffuse	8 (9.0)	13 (22.8)	
	Mixed	8 (9.0)	15 (26.3)	
	Unknown	59 (66.3)	10 (17.5)	
PD-1 (%)	Negative	18 (20.2)	47 (82.5)	<0.001
	Positive	6 (6.7)	10 (17.5)	
	Unknown	65 (73.0)	0 (0.0)	
PD-L1 (%)	Negative	8 (9.0)	34 (59.6)	<0.001
	Positive	16 (18.0)	23 (40.4)	
	Unknown	65 (73.0)	0 (0.0)	

BMI, body mass index; PD-1, programmed death 1; PD-L1, programmed death-ligand 1; ICIs, PD-1/PD-L1, inhibitors.

**TABLE 5:** The blood parameters for treatment (ICIs and chemotherapy).

n	Level	ICIs 89	Chemotherapy 57	*p*
GIPI (%)	Good	59 (66.3)	47 (82.5)	0.052
	Intermediate/poor	30 (33.7)	10 (17.5)	
LDH (%)	<250	73 (82.0)	51 (89.5)	0.322
	≥250	16 (18.0)	6 (10.5)	
dNLR (%)	<3.0	69 (77.5)	53 (93.0)	0.026
	≥3.0	20 (22.5)	4 (7.0)	
WBC (%)	<6.44	38 (42.7)	35 (61.4)	0.042
	≥6.44	51 (57.3)	22 (38.6)	
NEU (%)	<3.82	37 (41.6)	36 (63.2)	0.018
	≥3.82	52 (58.4)	21 (36.8)	
LYM (%)	<1.70	44 (49.4)	29 (50.9)	1.000
	≥1.70	45 (50.6)	28 (49.1)	
MONO (%)	<0.48	36 (40.4)	36 (63.2)	0.012
	≥0.48	53 (59.6)	21 (36.8)	
EOS (%)	<0.09	44 (49.4)	23 (40.4)	0.366
	≥0.09	45 (50.6)	34 (59.6)	
BASO (%)	<0.02	18 (20.2)	16 (28.1)	0.372
	≥0.02	71 (79.8)	41 (71.9)	
RBC (%)	<4.34	40 (44.9)	32 (56.1)	0.250
	≥4.34	49 (55.1)	25 (43.9)	
PLT (%)	<232.0	45 (50.6)	27 (47.4)	0.836
	≥232.0	44 (49.4)	30 (52.6)	

ICIs, PD-1/PD-L1, inhibitors; GIPI, gastric immune prognostic index; LDH, lactate dehydrogenase; dNLR, derived neutrophil to lymphocyte ratio; WBC, white blood cell; NEU, neutrophils; LYM, lymphocyte; MONO, monocyte; EOS, eosinophils; BASO, basophil; RBC, red blood cell; PLT, platelet.

**FIGURE 2:**
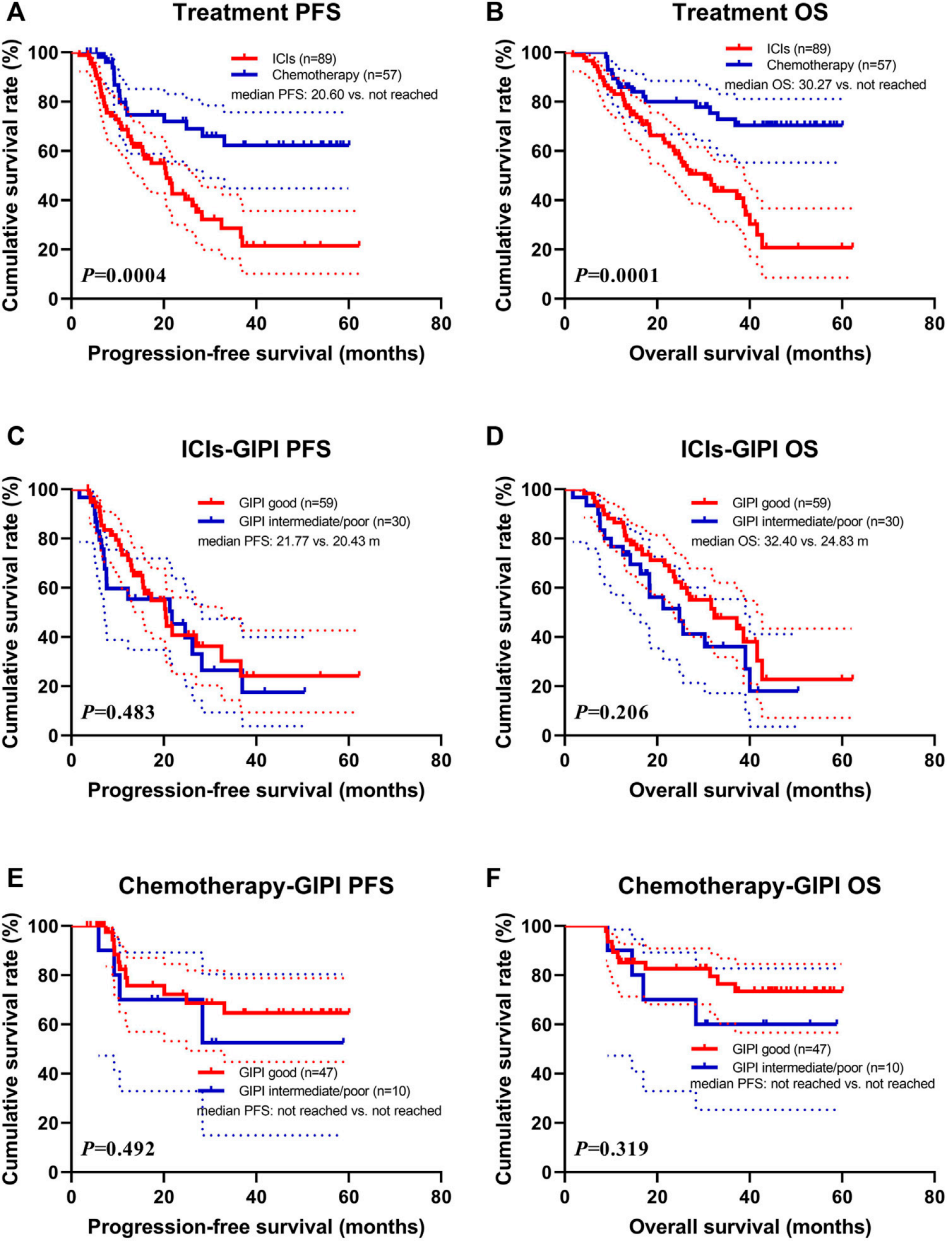
Survival according to ICIs and GIPI groups for **(A)** progression-free survival (PFS) by treatment; **(B)** overall survival (OS) by treatment; **(C)** PFS by ICIs; **(D)** OS by ICIs; **(E)** PFS by chemotherapy; **(F)** OS by chemotherapy.

Patients with PD-1/PD-L1 inhibitors treatment were associated with shorter PFS (median: 20.60 months vs. not reached; *p* = 0.0004) and OS (median: 30.27 months vs. not reached; *p* = 0.0001) than those with chemotherapy treatment (Figures 2A,B). In the ICIs group, patients with GIPI intermediate/poor were associated with shorter PFS (median: 20.43 vs. 21.77 months; *p* = 0.483) and OS (median: 24.83 vs. 32.40 months; *p* = 0.206) than those with GIPI good (Figures 2C,D). In the chemotherapy group, patients with GIPI intermediate/poor were associated with shorter PFS (median: not reached vs. not reached; *p* = 0.492) and OS (median: not reached vs. not reached; *p* = 0.319) than those with GIPI good (Figures 2E,F).

### Programmed Death 1/Programmed Death-Ligand 1 Only Positive Expression

**FIGURE 3:**
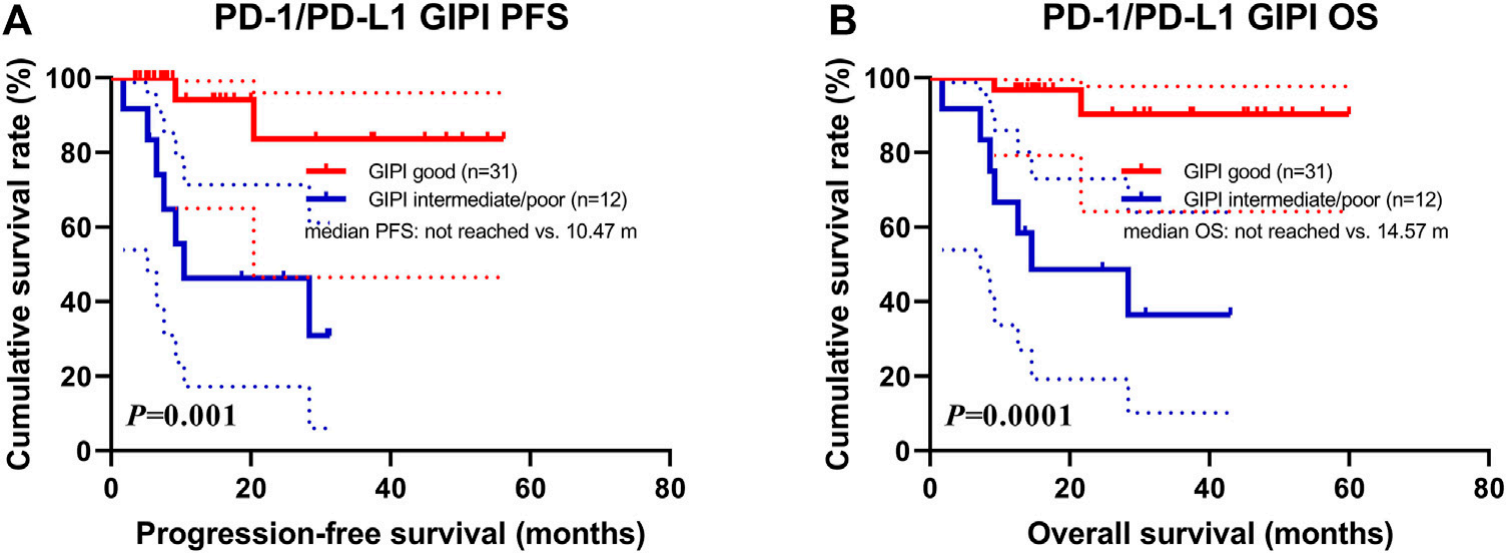
Survival according to PD-1/PD-L1 positive expression groups for **(A)** progression-free survival (PFS) and **(B)** overall survival (OS).

Data for PD-1/PD-L1 expression were analyzed on tumor cells using immunohistochemistry, according to standard practice. Expression of at least 1% was considered positive (Zayac and Almhanna, 2020). PD-1 status was positive in 16 patients (11.0%), negative in 65 (44.5%) and unknown in 65 (44.5%). PD-L1 status was positive in 39 patients (26.7%), negative in 42 (28.8%) and unknown in 65 (44.5%). Overall, PD-1/PD-L1 status was positive in 43 patients (29.5%), negative in 38 (26.0%) and unknown in 65 (44.5%). The high rate of missing PD-L1 status was because it was not mandatory for ICI prescription. According to the PD-1/PD-L1 positive status for 43 patients, 31 patients were GIPI good and 12 patients were GIPI intermediate/poor. According to the subgroup analysis, patients with GIPI intermediate/poor were associated with shorter PFS (median: 10.47 months vs. not reached; *p* = 0.001) and OS (median: 14.57 months vs. not reached; *p* = 0.0001) than those with GIPI good (Figures 3A,B).

## Discussion

Although the accuracy of gastric cancer treatment has been significantly improved in recent years, gastric cancer is still challenging (Smyth et al., 2020). ICIs, such as PD-1/PD-L1 inhibitors, have emerged as a promising treatment approach with curable potential and durable survival. However, many patients with gastric cancer receiving PD-1 or PD-L1 inhibitor treatment do not experience survival benefits due to substantial heterogeneity (Akin Telli et al., 2020; Kawazoe et al., 2021). Biomarkers, including PD-1, PD-L1, CTC, and TMB, have limited predictive accuracy due to the unavailability of tumor tissue and molecular or microscopic analyses (Lianidou et al., 2015; Yi et al., 2018; Ritterhouse, 2019). Systemic inflammatory status has been found to be related to the survival and prognosis of patients with different types of cancer (McMillan, 2009; Diakos et al., 2014). Although the inflammatory markers have been observed in patients treated with surgery, chemotherapy, or targeted therapy, the effect of systemic inflammatory status on immunotherapy benefit is not well known (Clarke et al., 2011; Li et al., 2020; Ravindranathan et al., 2021). Hence, it is important to look for biomarkers that can predict treatment outcomes.

The LIPI, based on dNLR and LDH, was first developed by Mezquita and colleagues and is supposed to be related to ICIs outcomes in patients with non-small cell lung cancer (Mezquita et al., 2018). The prognostic relationship between higher LIPI scores and poorer outcomes has also been confirmed in lung cancer. Most notably, the LIPI is an ideal biomarker because it is non-invasive, cost-effective, and can easily be obtained from serum. More recently, in a monocentric retrospective cohort of 720 advanced melanoma patients treated with ipilimumab, dNLR ≥3 was associated with a negative effect on survival and may help in risk-group stratification and disease-management strategies (Ferrucci et al., 2016). LDH is a classic inflammatory marker in cancer patients and has been found to be related to shorter survival when increased from 1 to 2.5 ULN (Van Wilpe et al., 2020). Diem and colleagues reported that LDH was a useful biomarker at baseline and during treatment to predict objective response in 66 consecutive patients with advanced or metastatic melanoma treated with nivolumab or pembrolizumab. It was significantly associated with shorter OS, reflecting the potential value of monitoring these markers (Diem et al., 2016). Castello and colleagues have reported immune-metabolic prognostic index (IMPI) at the first restaging, combining both inflammatory and metabolic biomarkers, was correlated with PFS and OS. IMPI can be a potentially valuable tool for identifying NSCLC patients who are likely to benefit from ICI (Castello et al., 2020).

To our knowledge, this is the first study to investigate the relationship between GIPI and survival outcomes of gastric cancer patients undergoing PD-1/PD-L1 inhibitors treatment. Our results indicated that patients in GIPI good group treated with PD-1/PD-L1 inhibitors have improved PFS and OS compared with those in the GIPI intermediate/poor group. The prognostic value of this biomarker was also consistent with that of previous studies for patients who received ICIs therapy in advanced hepatocellular carcinoma (Chen et al., 2020), advanced small cell lung cancer (Li et al., 2021), esophageal squamous cell carcinoma (Feng et al., 2021). Furthermore, a dNLR >3 and LDH >250 U/L were associated with shorter PFS and OS. Based on the univariate analysis, our findings also showed that GIPI was related to PFS and OS in gastric cancer patients who received PD-1/PD-L1 inhibitors therapy. However, the multivariate analysis indicated that GIPI was not the potential independent prognostic factor for PFS and OS. Nevertheless, considering the retrospective nature of this study, the negative results of PFS and OS should be interpreted with caution as they may have been influenced by multiple factors, including the enrolled patients and tumor type. In contrast, the difference in PFS and OS between GIPI good group, and GIPI intermediate/poor group is more convincing. Simultaneously, we also analyzed the difference by a treatment that was significant. Furthermore, we conducted a subgroup analysis by ICIs or chemotherapy, and the results showed that the patients with GIPI intermediate/poor were associated with shorter PFS and OS. We also performed the PD-1/PD-L1 expression status by GIPI, and the results indicated patients with GIPI intermediate/poor were associated with shorter PFS and OS.

There are several plausible mechanisms to evaluate the relationship between GIPI and the prognosis of gastric cancer. Neutrophils can be influenced and manipulated, including the differentiation process and the development of different phenotypes and functional polarization states (Shaul and Fridlender, 2019; McFarlane et al., 2021). In the proinflammatory state, this will induce “emergency granulopoiesis” that rapidly increases the production of neutrophils, thereby releasing immature or poorly differentiated neutrophils related to tumor progression (Zhang et al., 2020). The increase in LDH level is the product of tumor glycolytic activity and tumor necrosis caused by hypoxia, and the latter is related to the high tumor burden (Van Wilpe et al., 2020). The LDH levels are inversely associated with response to checkpoint inhibitors and glycolysis inhibitors (Laganá et al., 2019; Ke et al., 2021).

This study had several limitations. First, our exploratory evaluation was retrospective, and the data was from a single-center study conducted on a small number of gastric cancer patients. As a result, the sample size of the GIPI poor group was too small. Hence, we divided the study subjects into two groups (GIPI good and GIPI intermediate/poor) rather than three groups (GIPI good, GIPI intermediate, and GIPI poor). In addition, some confounding factors and selective bias could not be avoided. Second, most of the enrolled patients received PD-1/PD-L1 inhibitors as their second-line treatment or beyond. The degree of baseline inflammation may be affected by previous treatment. More so, due to the bias of drug selection, the results should be interpreted with caution. Despite these limitations, a unique aspect of this study was the combined model of three baseline peripheral blood markers for the outcome of PD-1/PD-L1 inhibitors. Finally, GIPI is a nonspecific tumor marker; hence, the need to further verify the correlation between GIPI and cancer prognosis in a prospective study.

## Conclusion

In conclusion, based on the GIPI intermediate/poor group, combining dNLR >3 and LDH > ULN was related to poor outcomes. The GIPI may be useful for identifying gastric cancer patients who are unlikely to benefit from treatment. The GIPI is also related to the PD-1 or PD-L1 expression, and poor baseline GIPI correlated with poor outcomes for PD-1 or PD-L1 expression status.

## Data Availability

The raw data supporting the conclusions of this article will be made available by the authors, without undue reservation.

## Abbreviations

dNLR, derived neutrophil to lymphocyte ratio; LDH, lactate dehydrogenase; GIPI, gastric immune prognostic index; ICIs, immune checkpoint inhibitors; ULN, upper limit of normal; PD-1, programmed death 1; PD-L1, programmed death-ligand 1; OS, overall survival; PFS, progression-free survival; LIPI, lung immune prognostic index; WBC, white blood cell; NEU, neutrophils; LYM, lymphocyte; MONO, monocyte; EOS, eosinophils; BASO, basophil; RBC, red blood cell; PLT, Platelet.
